# Target-the-Two: a lab-in-the-field experiment on routinization

**DOI:** 10.1007/s00191-022-00795-2

**Published:** 2022-12-01

**Authors:** Giuseppe Attanasi, Massimo Egidi, Elena Manzoni

**Affiliations:** 1 Department of Economics and Law, Sapienza University of Rome, Rome, Italy; 2 GREDEG, CNRS, Université Côte d’Azur, Valbonne, France; 3 School of Government, LUISS University, Rome, Italy; 4 Department of Economics, University of Bergamo, Bergamo, Italy

**Keywords:** Creativity, Routinization, Target-The-Two game, Lab-in-the-field experiment, C93, D91, O31

## Abstract

The paper investigates the cognitive determinants of routinization and creativity by means of a lab-in-the-field experiment run at the 20th edition of a mass gathering festival in Italy (“La Notte della Taranta”). Subjects play repeatedly the puzzle version of the Target-The-Two game (32 hands). In hands 1-16, the strategy that is optimal given the card distribution is always the same and it is the easiest to be discovered. Conversely, in hands 17-32, subjects are exposed to games where the optimal contextual strategy may differ from the one with which they have been made familiar. We investigate whether and how, in hands 17-32, subjects remain routinized on the familiar strategy, or creatively choose a different one. We define as “experts” those subjects who played the optimal contextual strategy in the overwhelming majority of hands 1-16. In hands 17-32, we find several subjects playing the familiar strategy even when it is not the optimal one, regardless of whether they are experts or not. This shows that routinization is deep-rooted in the cognitive individual process. Furthermore, routinization pays off only for inexpert subjects: creative inexpert subjects are slower and they fail to find the optimal contextual strategy in several hands. Among expert subjects instead, creative subjects, although still slower, need less moves than routinized ones to find the optimal contextual strategy.

## Introduction

In the recent years, economics has started considering how creativity affects economic outcomes, how we can measure it, and what can be done to promote its development.

A relevant part of the literature dealing with creativity recognizes the role that individual characteristics, such as genetics, divergent thinking and cognitive development, play in fostering it (see, e.g., Simonton 2000).

To study creativity, and its origins, however, we need to agree on a definition of creativity. From a psychological point of view, creativity has been seen as an innate feature of individuals, while from a social perspective, it is acknowledged that many external factors can influence it. Measuring creativity and identifying creative paths in the laboratory is one of the most recent challenges in experimental economics (see, e.g., Charness and Grieco 2019). However, reliable measures of creative and innovative abilities in real-life situations have not been yet produced by the experimental literature in either psychology and economics (see the surveyed articles in Attanasi et al. 2021a).

Our research aims to fill this gap by considering a specific aspect of creativity, that is the ability of subjects to explore the space of possible solutions to a problem. In this respect, creativity is opposed to routinization, in the sense that routinized subjects maintain their routines and do not test alternative procedures that may prove to be more efficient than the adopted one (or not).

To do so, we have run an experimental study aimed at measuring creativity and routinization in the field and to detect correlations between attitudes to routinization on the one side, and subjects’ cognition and other idiosyncratic features on the other side. Specifically, in this study we explore the causes and effects of creativity and routinization by means of a lab-in-the-field experiment, with a twofold aim. First, we aim to test the formation of routines outside the lab (lab-in-the-field), and the causes of routinization, by looking both at individual characteristics (gender, age and education) and individual behavior (such as alcohol consumption). Second, we investigate the effects of routinization and creativity on performance, in order to understand when it is the case that creative subjects outperform routinized ones, or vice-versa.

We run the experiment with a mobile lab positioned at the 20th edition of the traditional music Festival “La Notte della Taranta”, in Italy. To this aim, we have also developed a web platform – Graphgames –﻿, which was a necessary condition to implement the field experiment with tablets and analyze creativity and routinization in real time. We proposed to subjects a ‘puzzle’ game experiment consisting in a repeated individual decision problem, namely the one-player version of the *Target-The-Two* game (Cohen and Bacdayan 1994). The Target-The-Two game admits two strategies that can be optimal, depending on the initial configuration. The experiment is composed by a maximum of 32 hands of the game, where in the first 16 hands (training phase) subjects face only problems that are all optimally solved by one of these two strategies. In the last 16 hands, instead, configurations are selected randomly, and therefore, on average, half of them can be optimally solved with one strategy and the other half with the alternative one.

The two-phase structure of the game allows us to investigate both the learning process, i.e., the ability of understanding and adopting the optimal strategy in the first phase, where all hands have the same optimal solution, and the routinization process in the second phase, i.e., the process for which agents keep using the same routine (strategy) even when they face tasks that possibly have different optimal solutions. Players who after the training phase routinized on one strategy stick to it once they have identified it. Creative players, instead, explore different strategies according to the specific configuration of the game, possibly selecting in each hand the strategy that would let them solve the game in a more efficient way, i.e., with a smaller number of moves.

We explore the possibility that creativity and routinization may have different effects on agents depending on their level of expertise. We measure expertise as the ability to learn and adopt the optimal strategy in the first phase, where subjects are trained with a series of problems that are all solved optimally with the same strategy. We find that for expert subjects overall performance does not differ between creative and routinized subjects, even though creative subjects seem to make a lower number of moves, and routinized subjects are faster. Things are different when focusing on inexpert subjects. For inexpert subjects routinization improves performance significantly. The channel of the improvement is twofold: on the one hand routinization reduces variance in the performance, on the other hand it reduces the average time by making subjects faster.

The contribution of our paper is threefold. First, we provide a measure of one particular aspect of creativity as opposed to routinization, thus contributing to the literature on experimental measures of creativity (see Attanasi et al. 2021a for a comprehensive survey of the literature).

Second, we contribute to the literature on the cognitive determinants and the effects of routinization, and on the link between expertise and routinization. From previous experiments (Luchins and Luchins 1959) we know that the more a player becomes expert, the more his reaction becomes fast and automatic. We explore this link further, by investigating the interaction between the level of agents’ expertise and the efficiency of routinization, which is a novel contribution in the literature. Moreover, we contribute to the specific literature on the Target-The-Two game and routinization, which is extensively discussed in Section 2, by implementing the puzzle version of the Target-The-Two game so as to isolate learning and routinization that are not related to coordination issues but rather to the cognitive individual process. In this regard, our study departs from existing work in the literature (e.g., Egidi 1996, Egidi and Narduzzo 1997, Garapin and Hollard 1999, and Wollersheim and Heimeriks 2016, with the exception of Egidi 2016).

Finally, we contribute to the literature on lab-in-the-field experiments, i.e., studies conducted in a naturalistic environment targeting the theoretically relevant population but using a standardized, validated lab paradigm. The “lab-in-the-field” methodology combines elements of both lab and field experiments in using standardized, validated paradigms from the lab in targeting relevant populations in the field (Gneezy and Imas 2017). On the one side (lab), employing a standardized paradigm permits the experimenter to maintain tight control; on the other side (field), targeting the relevant population and setting increases the applicability of the results. Indeed, our study is the first lab-in-the-field study of the Target-The-Two game, with a sample of 480 participants. The gathering event where we carried it out is “La Notte della Taranta” Festival, held each year since 1998 in the province of Lecce (South of Italy) in late August (www.lanottedellataranta.it/en/). The event is among the most important European folk festivals: in the last 10 years, it was able to attract approximately 300,000 attendees on average per year. Our experiment was run during the rehearsal of the final concert (50,000 attendees) and the final concert (100,000 attendees) of the 20th Edition (August 2017). Thus, the gathering event guaranteed high population size and heterogeneity (more than half of the attendees being non-local, tourists coming from almost all Italian regions, age from 14 to over 60, education from primary school to PhD, etc.), with the distribution of the main idiosyncratic features being representative of the Italian population (see Attanasi et al. 2017).

However, our lab-in-the-field experiment is different in spirit to what Harrison and List (2004) term as *artefactual field experiment*, which they define as “$\ldots$the same as a conventional lab experiment but with a nonstandard subject pool.”^1^ In line with Charness et al. (2013), we argue that the physical location of the lab is not what defines a method, and laboratory experiments that are run outside of the university are not best described as field experiments. In this regard, the gathering festival that we have chosen for our lab-in-the-field study had a second important feature: the small village where the event was held was transformed for two nights into a huge dance floor with musical contamination, prevalence of non-local attendants, and, more importantly, plenty of collateral events of different nature (social, artistic, entertainment) taking place in the squares around the main stage. Our experiment was framed as one of these events, as a series of tournaments taking place in a gazebo placed at about 300 meters from the main stage, the first tournament starting on the afternoon of the rehearsal of the final concert and the final tournament ending after the end of the final concert (more than 24 hours later). It was advertised in local newspapers and TV news some days before the concert, and across the concert through posters, flyers, and announcements on social networks. With this, although our subjects were aware of participating in an experiment, the naturalistic environment were it was implemented made it seem much more like a game of competition with cards, in the true spirit of the Target-The-Two game of Cohen and Bacdayan (1994).

The rest of the paper is organized as follows. Section 2 introduces the Target-The-Two game, which is at the heart of our experiment. Section 3 describes the experimental design and states the experimental hypotheses. Section 4 presents the results and Section 5 concludes.

## Target-The-Two

**Fig. 1:**
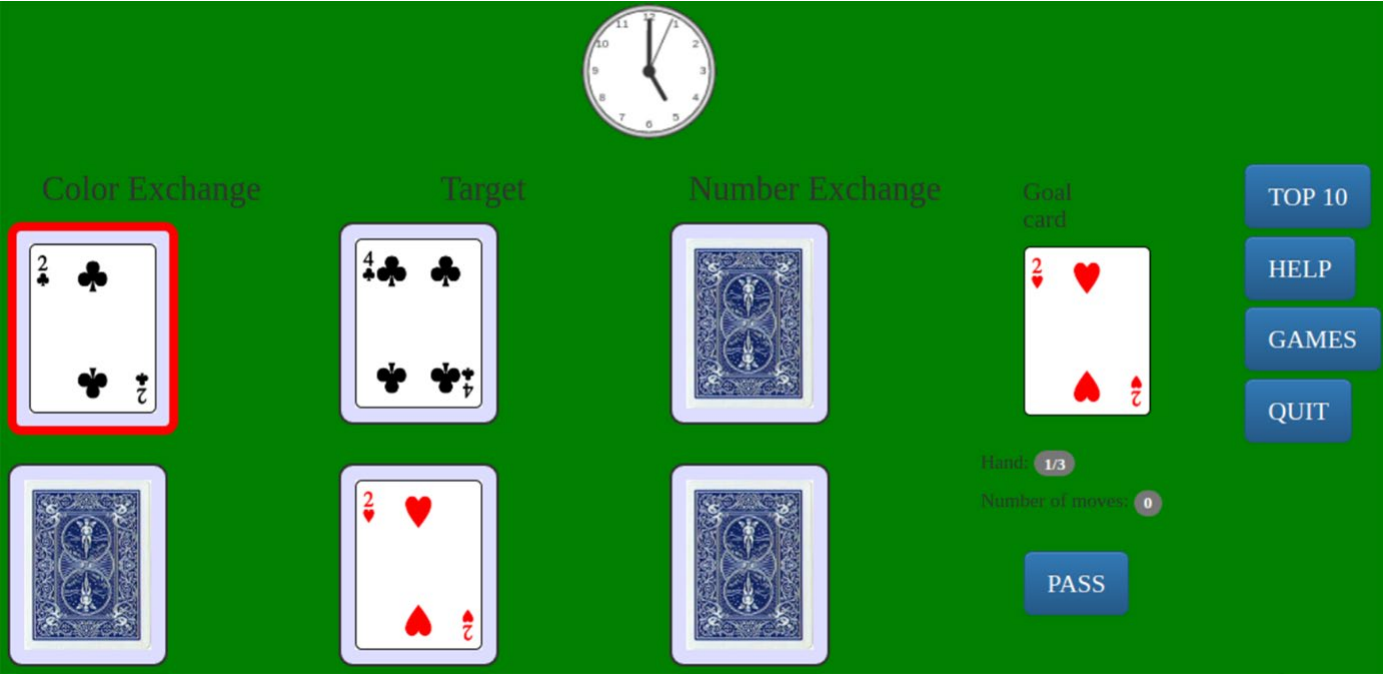
Target-The-Two Game: a deck. The button “Top 10” allows the participant to look at the top 10 performances at the end of the game. The button “Help” can be used before or during the game to check the rules. The button “Games” is used by the experimenter before the game to attribute the subject to a specific treatment. The button “Quit” can be used to leave the experiment before the end

Target-The-Two (TTT henceforth) is a well-known card game in the field of behavioral and experimental economics. It was first introduced by Cohen and Bacdayan (1994) to study experimentally the behavioral routinization of pairs of card players who cooperate to achieve a common goal. Its properties in terms of detection of human cognition and decision processes have been extensively highlighted by Egidi and Narduzzo (1997).^2^ TTT is our prototypical game for the following fundamental feature: it has a simple enough mathematical representation while being interesting and challenging for people (see Fig. 1, which represents a deck of our experimental interface). It is appropriate for lab-in-the-field experiments, as on the one hand it is easy to explain to subjects who are not familiar with experiments or with economic language, and on the other hand it is sufficiently rich to allow an interesting analysis of decision making, routinization and creativity.

### TTT as a coordination game

TTT requires the use of six cards (numbers 2, 3 and 4) and two distinct seeds: Hearts (H) and Clubs (C), and it can be played as a cooperative two-player card game, or in a puzzle form (single player). The aim of the game is to put a goal card in the target position. For half of the treatments, and for the sake of this theoretical description of the game, we let the goal card be the 2 of Hearts (2H). In its original form, with two distinct players, the players are called Color-Keeper and Number-Keeper. When cards are dealt, they are positioned in two rows of three cards each. In the top row we have the card in the hand of Color-Keeper, the target, and the card in the hand of Number-Keeper. In the bottom row we have three cards, two of which are covered (left and right), and one facing up (the central one, under the target position). Each player can see only the card in his hand, the card in target and the card facing up (under target). In each hand, the objective of the game is to put the goal card into the “Target” position. A move is any exchange of the player’s card with one of the other board card (except for the other player’s card) or “PASS”: passing the turn. Players’ moves to the target position are constrained as follows: Color-Keeper can exchange the card in his hand with the target card only when they are of the same color, while the Number-Keeper can exchange the two cards only when they are of the same number.

**Fig. 2:**
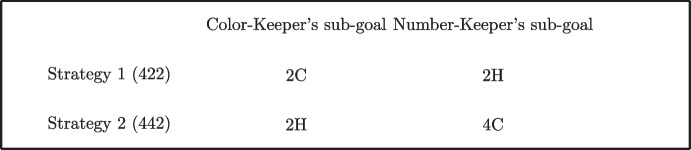
Key-cards (i.e., sub-goals) for coordinated strategies

If we exclude some very elementary card distribution (which we do not use in the experiment), the TTT game admits two different strategies to achieve the goal, each of which is optimal for a different subset of the initial card distributions (Egidi 1997). Using the deck of Fig. 1 as example, the first strategy requires first Color-Keeper to search and put in target 2C, and then Number-Keeper to search and put in target 2H; the sequence of cards on target is therefore 4C-2C-2H (hereafter 422). The second strategy, 442, requires first Number-Keeper to search and put in the target 4H, then Color-Keeper to search and put in the target 2H; the sequence of cards on target is therefore 4C-4H-2H (hereafter 442). The two strategies are reciprocally incompatible, because either Number-Keeper puts the 2H in target (422) or Color-Keeper puts 2H in target (442). Therefore, in the two-player version of the game, coordination is strictly necessary. Coordination occurs because each strategy defines two compatible sub-goals for the pairs, i.e., two different key-cards which trigger their actions in a coordinated way (Fig. 2).

In the particular case of Fig. 1 it is clear that the first strategy (422) is more efficient than the second one (442), but of course there are different card distributions which reverse the situation by making the 442 strategy more efficient than 422. Both strategies are applicable when the card in the target at the beginning are 3C or 4C (as in Fig. 1). In any other case the hand can be solved by one player only: if 2C is in the target, only Number-Keeper moves (his sub-goal of 422); if 3H or 4H are in the target, only Color-Keeper moves (his sub-goal of 442).

Experiments show that, if the game is played repeatedly, pairs of players jointly learn at least one strategy and become familiar with it (Egidi 1996, Egidi and Narduzzo 1997, Garapin and Hollard 1999, Egidi 2001, Wollersheim and Heimeriks 2016). When they apply the strategy they have learnt, the pairs are clearly acting in a strongly coordinated way; this means that each member of a pair has identified the key-cards of the strategy and makes the action required to realize his sub-goal within the joint target (see Fig. 2). At this point of the tournament the execution of a strategy becomes largely automatic because the key-cards trigger the actions of the pair in a coordinated way.

### Routines and creativity

Cohen and Bacdayan (1994, p. 555) define organizational routines as “patterned sequences of learned behavior involving multiple actors who are linked by relations of communication and/or authority”. They consider the occurrence of repeated sequences of actions to be the most salient feature of routinized behaviors. As a consequence, in order to verify if an individual’s actions fit to a routine, we need to check whether the sequences of actions are repetitive: in our case this means that players are supposed to react with the same sequence of actions if the same distribution of cards appears on the board again. To check routinization according to this definition, we would need to set up an experiment where the same card distributions are presented two or more times: on top of making the design of the experiment heavier, this structure of the experiment could skew the test results by inducing players to biased behaviors, in a situation where there are two alternative strategies.

We can instead verify the application of a strategy in a more general and accurate way simply by comparing the sequences produced by the pairs with the sequences produced according to the division in sub-goals sketched in Fig. 2. This means that we do not consider only the sequence of actions but the sequence of rules that a pair must use to produce the actions. In general we can completely define player’s behavior through condition-action rules, and in consequence the routines will be detected by checking if players’ action patterns are fitting with the action sequences generated by a system of condition-action rules.

Moreover, this approach allows us to measure the degree of routinization of a pair of players in a precise way. According to March and Simon (1958), routinization exists when players do not modify their behavior even when the decision context changes. According with this view (and with the findings of Luchins and Luchins 1959), a player who, after the discovery of one strategy, continues to use it for the rest of the tournament even when a better strategy exists, can be defined “routinized”. Therefore, if we could extend the findings of March and Simon (1958) and Luchins and Luchins (1959) from the individual to the collective learning process, then we could say that a pair is routinized when both players are locked in one strategy only, and that they cannot discover and use the alternative one when it is more efficient.

As a consequence, in this context we consider creativity as measured by the ability to explore widely the space of the game configurations: players who remain locked in one strategy do not actively explore the space of configurations while creative players are able to get out from the lock-in and discover a new strategy. In this regard, we interpret the TTT game as a “closed creativity” task, i.e., a creativity task where ex-ante goals and constraints are imposed, which allows to study creativity as opposed to routinization. Comparing creativity and routinization in a way that allows us to distinguish them clearly is not easy with other closed creativity tasks proposed in the literature of psychology and economics, e.g., packing and anagrams (Ariely et al. 2009), algorithms (Boudreau and Lakhani 2011), scrabbles (Eckartz et al. 2012) and scrabble games (Brüggemann et al. 2016).^3^

Therefore, our puzzle version of the TTT game is meant to detect the intrinsic lock-in of routinization and to further explore its causes, thereby allowing to better understand some features of human creativity: while creativity is generally considered as the ability to find new solutions to a problem, we suggest a specific definition, i.e., we measure it as the capability to actively get out from the lock-in, which is strictly related to the classical dichotomy between exploration and exploitation (March 1991) or between static vs. dynamic capabilities (Benner 2009) in organization theory.^4^

### Routines and automaticity

An interesting question emerges from the features of TTT players’ behavior: can the process of “routinization” be attributed only to the players’ cooperative interaction or it depends also on the individual cognitive process during the search for a strategy? Cohen and Bacdayan (1994) assume that routinized individual behaviors are stored as procedural memory, a property which directly relates to the opaque nature of the knowledge embodied in routinized behaviors and their partially inarticulate nature. Their view suggests that the automaticity with which players repeat the same sequences of actions can be explained in terms of automaticity in their mental processes. Studies on the mechanization of thinking – the so-called “Einstellung effect” – have a long tradition in psychology (Luchins 1942, Luchins and Luchins 1950). The literature has suggested that routinized behaviors are based on “routinized thinking”, i.e., on the automatic use of “chunks” which enable individuals to save on mental effort (Newell and Simon 1972, Laird et al. 1987, Newell 1990). The experiments by Luchins and Luchins (1959) lead to the conclusion that the routinized behaviors can be explained in terms of bounded rationality or more precisely in terms of the dual model account of reasoning.^5^ According to this model, when players have discovered one strategy, the key elements of this familiar strategy will come automatically to their mind (i.e., become more accessible) for the following hands of the game; accessibility then governs players’ attention making the application of the familiar strategy easier than the search for a new one. Our hypothesis is that the routinized behavior of a pair in the TTT game could be explained as originated also by the cognitive features of the individual learning process.

### TTT as puzzle for testing creative abilities

Simon (1969, p. 54) states that “problem solving is often described as a search through a vast maze of possibilities, a maze that describes the environment. Successful problem solving involves searching the maze selectively and reducing it to manageable proportions.” According to Simon (1969), human problem solving is a process that can be empirically observed and formalized: the search for a solution to a problem is represented as search in the network of possible alternatives. In the case of games, the alternatives are the different states of the game and the solutions are paths to the winning states. Given the enormous amount of alternatives that must be explored – even for simple games – the discovery of a new solution depends from the ability of a person to find a path to the solution; every individual may have different search criteria and different guiding principles, or “heuristics”, that come from their experience and their attitude to learn. As a consequence, restricting for a moment our definition to games, creativity can be considered as the ability to develop a search to the winning states, which requires an idiosyncratic ability to learn. This definition can be extended to the more general problem solving context, in the sense that creativity can be considered as the ability to find new solutions to a problem or, more importantly, new representations of a problem.

**Fig. 3:**
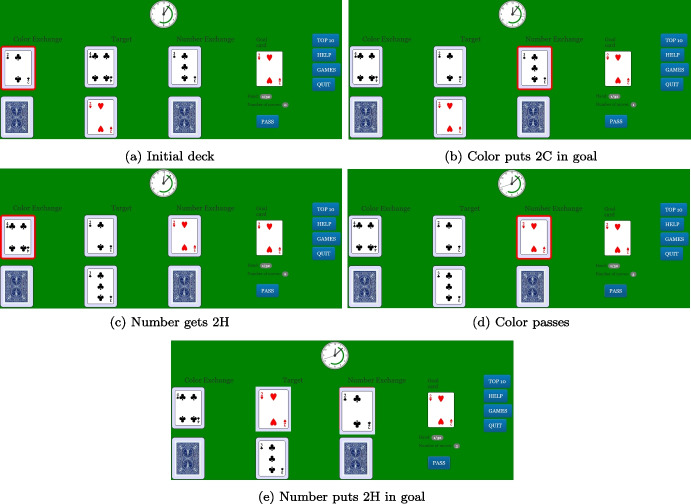
An example of TTT solo: a 422 play

To measure individual creativity, we implemented the TTT game in a puzzle form. In this form, the game has the same rules as in the original version, but with only one player who plays both roles in turn (Color-Keeper and Number-Keeper). Therefore, the constraints on card exchanges are the same, i.e., the card in position “Color” can be exchanged with the card in “﻿Target”﻿ only when the two cards have the same color, while the card in position “Number” can be exchanged with the “﻿Target”﻿ only when the cards’ number is preserved. In the game, the player sees both the card in position “Color” and the card in position “Number”. The player moves alternatively from each position according to a red frame that illuminates the position from which he has to move (in Fig. 3, the card in the top left corner for panels (a) and (c) and the card in the top right corner for panels (b) and (d)). Importantly, the TTT puzzle has exactly the same strategies and properties of the original game. Figure 3 shows an example of a deck in which strategy 422 is optimal, and it shows how the strategy is played out. The game starts with the configuration depicted in panel (a). The deck is optimally solved with strategy 422. Hence, at first the player puts 2C in goal from the Color position (panel (b)); then, playing as a Number player he gets 2H by exchanging 3C with 2H. We are now in the configuration of panel (c), where it is the turn of Color, who passes (panel (d)). Now the player, from the Number position, closes the game by exchanging 2H with 2C (panel (e)).

One of the he main features of this puzzle version of the TTT game is that – while maintaining all other characteristics of the original TTT game – having an individual task provides a cleaner measure of individual creativity/routinization, since – as we explain in the next sub-section – it constrains players to use cognitive abilities for the task solution only. Indeed, in our puzzle version of the TTT game it is possible to check experimentally both the discovery of a new solution within the same problem representation, and the discovery of a new representation; for every given goal, two different strategies to achieve it exist, and some players are flexible and able to get out from the first strategy they have learnt. We define these players as “creative”, in contrast to routinized ones.

### TTT as puzzle for isolating the cognitive determinants of the process of collective learning

Scholars in the field of strategic management have given particular relevance to identifying and testing various aspects of dynamic capabilities, broadly defined by Teece et al. (1997, p. 516) as “the firm’s ability to integrate, build, and reconfigure internal and external competences to address rapidly changing environments”. In relation to the routine interpretation of the organizations, dynamic capabilities have been defined by Zollo and Winter (2002, p. 340) as “a learned and stable pattern of collective activity through which the organization systematically generates and modifies its operating routines in pursuit of improved effectiveness”.

Cohen and Bacdayan’s (1994) TTT game, insofar it allows to study the discovery of “modes of cooperation” through which two players achieve a shared goal, is particularly interesting for understanding the creation of joint competences of the players, and the related emergence of organizational routines. Cohen and Bacdayan (1994) provided preliminary measures to assess whether the patterns of interaction between the subjects can be considered as routines, and showed that routines are stored in the procedural memory.

A sequence of papers have shed light on this process. Egidi (1996) was the first to replicate Cohen and Bacdayan’s (1994) work, by clarifying the definition of routines, based on his analysis on “production rules” of the form “If Condition then Action”. Egidi and Narduzzo (1997) have run the first systematic analysis of routinization in two-player TTT game, by identifying the 422 vs. 442 optimal strategies to which players routinize, which are the same two strategies on which our study focuses. They showed evidence of the path-dependent nature of routinized behaviors in cooperative contexts.

Garapin and Hollard (1999) have extended the previous studies by comparing different monetary incentives, with experimental gains negatively depending on the number of moves required to achieve the goal in one treatment, and on the amount of time required for hand’s completion in another treatment. These two are the same types of monetary incentives on which our experiment relies (together with the number of completed hands), fixed across treatments according to a lexicographic order. Garapin and Hollard (1999) found that subjects tended to play in a more “routinized” way under incentives on the amount of time, although this did not increase performance as compared to incentives on the number of moves. Their main concern was about subjects’ performance in the TTT game, which we present as a preliminary hypothesis to test in Section 3. They also proposed a definition of efficiency in the game based on time spent and number of hands played, which is close to the one that we use in our study. Furthermore, they were the first to provide results on the efficiency of learning a contextual strategy in the training phase, through routinization on the 422 and 442 strategy in the second phase. This is also a concern in our paper, where we extend their design of the training phase – they expose players to configurations that are more easily solved by the 422 strategy – by adding treatments where 442 is the optimal contextual strategy in the training phase.

More recently, Wollersheim and Heimeriks (2016) have implemented experimentally an extended version of the TTT game, in order to induce dynamic capabilities à la Teece et al. (1997) in the laboratory. After the initial training phase, they implemented a second phase where the two players’ roles (Color-Keeper and Number-Keeper) were exchanged and the goal card was changed and maintained fixed until the end of this phase. The second phase was meant to disentangle low vs. high dynamic-capability pairs according to the number of hands and moves per hand a pair required to respond to the environmental change. The experiment ended with a third phase where two pairs were merged into groups of four players based on their degree of dynamic capability. This study is close to ours in two features. First, in half of our treatments our goal card is changing in the second phase, although – differently from Wollersheim and Heimeriks (2016) – it also changes across hands of the second phase. Second, we also look at the comparison between players’ types in the second phase but – rather than assessing their dynamic capabilities – we focus on the dichotomy between routinized vs. creative players. As Wollersheim and Heimeriks (2016) found for players with high dynamic capabilities, we detect greater deliberation in action for those creative players who familiarized with the optimal contextual strategy in the training phase.

More in general, we highlight that– differently from ours– none of these experimental studies allows subjects to play the TTT game as single player, which eliminates strategic interactions between players by proposing an individual (rather than a strategic) task that constrains players to use cognitive abilities for the task solution only. This is relevant since the cognitive determinants of the process of collective learning are still imperfectly understood (Egidi and Sillari 2020). Our main question is therefore to verify if the cooperative construction of a joint strategy and the subsequent lock-in in the TTT game could be explained in terms of individual cognition, in line with what Luchins (1942) defines as “*Einstellung* effect”: after a preliminary phase where the same solution is optimal, most players’ performances become automatic, i.e., the prior knowledge triggers an automatic response to the given mathematical data, and players do not search anymore for alternative, possibly more efficient solutions (see the above sub-section “Routines and automaticity”).

Therefore, in the process of joint construction of a strategy and emergence of an organizational routine it is relevant to disentangle the mechanism of interaction among the two players from the cognitive aspects of the individual learning process. We make the assumption that after the emergence of routines, the lock-in is mostly due to the cognitive process of “mechanization of thought” discovered by Luchins (1942). While some preliminary indications that this assumption is correct come from the above mentioned laboratory experiments, a more extended and comprehensive work is necessary to better understand the cognitive aspects of the learning and routinization/creativity process, which is related to the symbolic manipulation of the items with which players make a representation of the game and its strategies.

In this regard, our experiment on a version of TTT where only one player makes the same job of the two cooperating individuals in the original TTT offers many advantages: by eliminating the strategic interactions within the pairs of players, it provides a clean identification of the individual features of the lock-in processes, and it allows to better understand the learning process, to detect the lock-in, and to further explore its cognitive determinants. More precisely, our puzzle version of the TTT game is meant to isolate the cognitive determinants of the process of collective learning, with a special focus on individual creativity, as discussed in the previous sub-section “TTT as puzzle for testing creative abilities”.

## Experimental design and hypotheses

This section describes the experimental procedures (Section 3.1) and design (Section 3.2), and then introduces and discusses the experimental hypotheses (Section 3.3)

### Procedures

The field experiment was run during the 20th edition of the traditional music Festival “La Notte della Taranta”, that takes place each year in August in the most southern part of the Apulia region (South of Italy). The event is among the most important European folk festivals, and attracted approximately 300,000 attendees per year during editions 2012-2017. It consists of 15 itinerant minor concerts (approximately 85,000 attendees per year, with a median of 7,000 attendees per concert) and a final concert (approximately 200,000 attendees per year). For both types of concerts entry is free. The data employed in our analysis was collected during the rehearsal of the final concert (Friday, August 24 2017: 50,000 attendees) and during the final concert itself (Saturday, August 24 2017: 200,000 attendees), where we observe a higher mass-gathering effect and a higher level of individual alcohol consumption. The final concert consists of a one-night huge dance floor, and its spectators come from every region of Italy (see Attanasi et al. 2013, 2019).

The sessions were run in a mobile laboratory (a gazebo), installed in the middle of the concert area, with the help of 15 experimental assistants who were continuatively in the field from Friday, August 23 2017 at 5 pm until 5 am of Sunday, August 25 2017. Each session had 20 participants, who played the experimental game on tablets. Each session had a 25 minutes time limit, and the best performer of each session was rewarded with 50€, so that the average payment was 2.5€. Overall, 480 subjects participated in the experiment. Table 1 reports summary statistics for our subject pool, which highlight heterogeneity as for socio-demographic variables.

The sample is slightly unbalanced as for gender (61% males) and it mostly consists of young adults: about 37% are 20 years old or younger, and 58% are between 20 and 40, average age being 24. More than 75% of them have completed high school, with 17% holding a bachelor degree and 5% having obtained a Master degree or a PhD. As for employment, more than half of the sample is represented by students, with the remaining 40% being composed mainly of employees and self-employed workers. As for provenance, more than 60% of the respondents are tourists, i.e., coming from a different area than the one where the mass gathering festival is held, mainly from other regions of Southern Italy, but also from Central and (especially) Northern Italy.

Since alcoholic drinks were extensively available during the festival, each player’s alcohol level was monitored before playing the experimental game. Specifically, we measured Blood Alcohol Concentration (BAC) through electronic breathalyzers (Testmed Safety digital professional alcoholometers).^6^ Table 1 reports a low average BAC = 0.09 g/l across the whole sample, explained by the fact that only 32.46% of our subjects were found with a positive BAC. Therefore, the overwhelming majority of the experimental participants were sober, while inebriated subjects reported an average BAC = 0.33 g/l, which is below the legal amount for driving established by the Italian legislation (0.50 g/l). However, when asked to guess their own BAC (before the measurement through the electronic breathalyzers) they report a belief that is significantly higher than the actual value, and this guess is four times larger when referred to the other festival attendees (belief others’﻿ BAC). The sizes of these two guesses – that we do not use in our analysis – show the relevance of the alcohol consumption in the perception of one’s own and others’ cognitive state when competing in the TTT game, thereby motivating our focus on the impact of BAC on game performance and routinization.

**Table 1:** Sample overview

	Mean	Std. dev.	Min.	Max.
Socio-demographic characteristics				
Female (D)	0.39	0.49	0	1
Age				
Up to 20 (D)	0.37	0.48	0	1
Between 21 and 40 (D)	0.58	0.49	0	1
Between 41 and 60 (D)	0.04	0.21	0	1
Over 60 (D)	0.01	0.07	0	1
Education				
No high school (D)	0.23	0.42	0	1
High school (D)	0.55	0.50	0	1
Bachelor (D)	0.17	0.38	0	1
Master/PhD (D)	0.05	0.20	0	1
Employment				
Student (D)	0.58	0.49	0	1
Employee (D)	0.20	0.40	0	1
Self-employed (D)	0.15	0.36	0	1
Unemployed (D)	0.05	0.21	0	1
Retired (D)	0.02	0.13	0	1
Provenance				
Locals (D )	0.36	0.48	0	1
Southern-Italian Tourists (D)	0.39	0.43	0	1
Central-Italian Tourists (D )	0.09	0.29	0	1
Northern-Italian Tourists (D)	0.15	0.36	0	1
Foreign Tourists (D)	0.01	0.11	0	1
Alcohol measurement				
BAC (alcohol test)	0.09	0.18	0.00	1.30
Belief own BAC	0.17	0.30	0.00	1.50
Belief others’ BAC	0.61	0.41	0.00	2.00

Dummy variables are marked with D

### Experimental design

In the experiment, subjects played repeatedly the TTT game in its puzzle (solo) version. We selected a set of starting configurations of the board, all of which were solvable with few moves if the same strategy (422 or 442) was played, while the alternative strategy (resp., 442 or 422) required a higher number of moves.

**Structure of the experiment** Before the beginning of the experiment, subjects were asked to fill in a questionnaire about their idiosyncratic features and to take an alcohol test, meant to measure their inebriation level (BAC) through electronic breathalyzers. Then, while waiting in line the end of the previous experimental session, they were distributed the experimental instructions, and invited to read them individually, with four experimental assistants ready to privately answer questions about the TTT game by the help of game posters attached to one of the sides of the mobile laboratory. Then, the subjects entered the mobile laboratory and sat in front of the tablet randomly assigned to them, with another experimental assistant who read publicly and aloud the instructions and answered questions about the TTT game publicly, by the help of a game poster attached within the mobile laboratory. Then, the experiment began. Subject faced a maximum of 32 hands of TTT in its solo version, and they had 25 minutes to complete the 32 hands, trying to minimize the number of moves needed to complete the task. The first 16 hands composed the *training phase*. The training phase is designed to guide the player to the best strategy to solve the provided hands, in a way that is unconscious to him (hidden and implicit training). In the training phase of the experiment, the player is exposed to configurations that are more easily solved by one of the two strategies (422 or 442) only, so to familiarize with it. In the *second phase* of the experiment, instead, the player faces a set of configurations where the optimal strategy is not constant: in the last 16 hands, configurations are selected randomly, and therefore, on average, half of the hands can be more easily solved (i.e., through less moves) with the 422 strategy and the other half with the 442 strategy.

**Treatments** The experiment has a 2 x 2 x 2 between-subject design, where the treatment variables are (i) the right contextual strategy to solve the game in the training phase (422 vs. 442); (ii) whether the opponent role’s cards are uncovered vs. covered; (iii) whether the goal card is fixed vs. goal card changes at each hand. We run 3 sessions per treatment, with a total of 60 observations for each treatment.^7^

*422 vs. 442 strategy.* The first treatment variable is the type of strategy (422 or 442) which is optimal in the training phase (first 16 hands) and on which subjects are therefore trained (in the example of Fig. 1, the optimal strategy is 422).

*Uncovered vs. covered cards.* The second treatment variable is whether the opponent role’s cards are uncovered or covered (in the example of Fig. 1, the opponent role’s cards are covered).

*Fixed vs. changing goal.* The third treatment variable is whether the goal card is fixed across different hands, or whether it changes with each hand (in the example of Fig. 1, the goal card is the two of hearts).

**Payment** In each session, composed by 20 subjects, the winner received 50€. The winner was selected according to the lexicographic order: (i) higher number of solved hands, (ii) smaller number of moves, (iii) lower amount of time.

We acknowledge that our payment scheme contrasts to other forms of incentives used with the TTT game. Indeed, standard payment schemes in this game usually involve loss functions in the form of an initial monetary endowment which is linearly reduced by imposing a fixed cost for each move – in resource-based contexts, or piece rate schemes involving a fixed gain for each completed hand within a fixed amount of time – in time-based contexts (see, e.g., Garapin and Hollard 1999). We adopted a payment scheme with a 1-winner high-stake tournament for two main reasons.

First, due to the “lab-in-the-field” nature of the experiment, paying a high amount of money (€50 prize) through a tournament to just 1 out of 20 participants made the payment scheme simple to understand, quick to implement, and highly incentivizing as for volunteer participation (for each session, we always had more than 20 subjects waiting in line and we never had to look for participants, given the big poster “You can win €50 in 25 minutes” on the top of the entrance to the mobile laboratory). The second reason relies on Charness and ﻿Grieco (2019) and several follow-up studies showing that, in individual “closed creativity” tasks, tournaments with monetary prizes to the winners boost individual creativity (see Attanasi et al. 2021a for a review on individual creativity and monetary incentives). Being the TTT game a closed creativity task, i.e., a task where ex-ante goals and constraints are imposed (as is usually the case for most of the economically relevant creative activity), we opted for a tournament with monetary prizes to the winner in order to boost individual creativity, given that we proposed the TTT game to subjects as a puzzle game experiment (individual decision problem).

### Experimental hypotheses

Let us first state two preliminary hypotheses that do not concern the routinization pattern, but only the effects that idiosyncratic characteristics of the players or of the game may or may not have on their performance. Recall that the winner in each experimental session of 20 participants was the player with the highest number of completed hands, where ties were broken by choosing the player with the lower (total) number of moves, and (if needed) the lower completion time. Therefore, we measure performance in three ways, which constitute the three levels of the above mentioned lexicographic order: number of hands completed, moves/hands ratio and time/hands ratio. The first is a direct measure of performance, while the other two indicators are higher the lower the performance of the player. In fact, our results show a $-0.79$ and a $-0.93$ Spearman’s rank correlation between number of hands completed and, respectively, moves/hands and time/hands ratio, both significant at the 0.1% level. From now on, with the term “*performance*” in the TTT game we mean all the three indicators: higher number of hands and lower moves/hands and time/hands ratios. This is in line with Garapin and Hollard’s (1999, p. 474) definition of efficiency as for carrying out “the same number of hands by a lower or equal number of moves and in a lower or equal total time”.

The first preliminary hypothesis extends the previous studies on subjects’ performance in the TTT game by looking at the relative use of the contextual strategies 422 and 442 (Garapin and Hollard 1999) within a richer set of changing environments, i.e., our 8 treatments. We begin by stating that we expect a worse performance in those treatments that are more complicated to play, i.e., those treatments with a higher cognitive load. In our experiment, we expect treatments with changing goal to be more complicated than treatments with fixed goal. The comparison between covered and uncovered treatments is less trivial: on the one hand the subject has to process less information in covered than in uncovered treatments; on the other hand, it is more complicated to figure out the optimal contextual strategy. Therefore, we expect the performance of covered treatments to be better in terms of average time, and possibly worse in terms of average moves. The total effect on the number of hands depends on which of the two aforementioned effects dominates. As for the strategy subjects are trained to, namely 422 vs. 442, given the symmetry of the two strategies, we expect no significant treatment difference. With this, we state H0.A as follows:

H0.A: (i) Performance is not affected by the strategy subjects are trained to. (ii) Treatments with changing goal have worse performance than treatments with fixed goal. (iii) Covered treatments bring higher moves/hands ratio and lower time/hands ratio than uncovered ones, with opposite effects on the number of hands.

The second preliminary hypothesis considers the relation between players’ individual characteristics and their performance. We have information on age, gender, education and inebriation level. As the task is implemented on young-friendly tablets, the higher familiarity with technology of younger (and possibly male) subjects should have a positive impact on their performance (see, e.g., Attanasi et al. 2021b, and references therein, as for higher videogame addiction by younger and male subjects). Thus, both age and female gender should have a negative impact on subjects’ performance. For gender, there is a reinforcement effect in the same direction: the experiment is designed as a tournament, and it is a common finding in the literature that females underperform in competitive environments (see, for example, Gneezy et al. 2003, and Gneezy and Rustichini 2004). All in all, we expect the performance of female participants to be lower than the males’ one. Furthermore, as the task requires logic reasoning, we expect education to have a positive effect on performance. For the same reason, we expect inebriation to have a negative effect on performance, as inebriation reduces deductive thinking (Gustafson and Nordlander 1994). With this, we state H0.B as follows:

H0.B: Performance is significantly worse for participants who are older, female, with low education or inebriated.

Now, before we discuss the experimental hypotheses on learning in the training phase and routinization in the second phase of the TTT game, we introduce our classification of the subjects in these two dimensions. First of all, we note that we cannot define a subject routinized on the basis of his behavior in the first 16 hands, as using the same strategy in the first 16 hands is the optimal behavior. Rather, the training phase allows us to investigate the learning process of the subjects, and their (acquired) expertise in the game. Therefore, we call *expert* those subjects who implemented the right contextual strategy during at least 75% of the hands of the training phase, and *inexpert* those subjects who did not manage to learn the optimal contextual strategy in the training phase, implementing it in less than 75% of the hands of this phase (i.e., less than 12 out of 16). To investigate routinization, instead, we have to focus on the second phase of the game, in which the optimal strategy varies across hands, so that we can distinguish subjects who routinize and always implement the same strategy (they have been previously made familiar with) from creative subjects who try to adapt their strategy to the specific problem they have to solve (despite having being previously made familiar with just one strategy). However, we note that not all subjects manage to reach the second phase. We call *early drop outs* those subjects who completed a number of hands strictly lower than 17, i.e., those subjects who did not complete hands in the second phase. Among those subjects who reach the second phase, we call (practically) *routinized* the subjects who in the second phase implemented the contextual strategy they have learned in the training phase for at least 75% of the hands completed in this phase. Finally, we call (practically) *creative* the subjects who in the second phase implemented the contextual strategy learned in the training phase in less than 75% of the hands completed in this phase.

We are now ready to start discussing our main experimental hypotheses. First of all, we expect both learning and routinization to be task-dependent (Ohly et al. 2006). Specifically, we expect learning to be lower in more difficult tasks. We instead expect routinization to increase in more difficult tasks for a twofold reason: first, it is more complicated for a subject to understand that a different strategy may be optimal in the new context; second, difficult tasks may induce more time pressure on the subject, thereby increasing routinization, consistently with the findings of Ohly et al. (2006). We can therefore state H1 as follows:

(i) Learning in the training phase and routinization in the second phase are both independent of the strategy subjects are trained in. (ii) Learning is higher in treatments with fixed goal and in uncovered treatments, and routinization is higher in treatments with changing goal and in covered treatments.

While we expect learning and routinization to be task-dependent, we expect them to be independent of personal traits such as age, gender and education. As a matter of fact, as shown in Chae and Choi (2019), correlation between routinization and age, gender or education is small and not significantly different from zero. There is instead evidence that alcohol consumption affects creativity and routinization. Norlander (1999) contains a review of the literature on the links between creativity and inebriation, and shows how alcohol may increase or decrease creativity depending on the specific type of creativity and on the phase of the creative process that we consider. We think that, in the literature discussed there, the most relevant result given our decision task is the one in Gustafson and Norlander (1994), who suggest that inebriation impair deductive reasoning. For this reason, we think that inebriated subjects should be less able than sober subjects to get expertise in the training phase and to routinize in the final phase. Therefore, we can state H2 as follows:

H2: Learning in the training phase and routinization in the second phase are both independent of personal traits (age, gender and education), and negatively dependent on the inebriation level of the subject.

The adoption of one strategy for all hands decreases the decision time of agents, by reducing the set of reasonable moves. As a consequence, it surely increases performance in the training phase, where expert subjects choose the correct contextual strategy faster. The effect of routinization on the second phase is more controversial. On the one hand, routinized agents are faster: they know where they have to go, since they are convinced that there is only one strategy to implement (the one they have been trained to); on the other hand, this is not always the right contextual one in the second phase, and therefore they might have a higher moves/hands ratio than creative subjects, who instead try to get the right strategy for each of the 16 hands of the second phase. Therefore, we formulate H3 as follows, by focusing on Garapin and Hollard’s (1999) measures of efficiency (moves and time):

H3: (i) Learning in the training phase is efficient: expert players perform better than inexpert ones. (ii) Routinization in the second phase has opposite effects on performance: routinized players are faster (lower time/hands ratio) but they have a higher moves/hands ratio than creative ones. If the first effect predominates, then routinization is efficient.

When testing H3, we will cross (i) with (ii) by comparing the performance of inexpert creative, inexpert routinized, expert creative, and expert routinized players. This latter analysis is in line with the analysis of Wollersheim and Heimeriks (2016), which used the TTT game to investigate the ability to develop a dynamic capability in the game and to assess the efficiency of players who managed to develop this dynamic capability in the game (in comparison with those who did not). Focusing on the second phase of the experiment, we instead investigate the efficiency of players who managed to get out from the first-phase lock-in and discover a new strategy (creative) in comparison with those who did not (routinized), in the light of the expertise acquired in the first phase of the experiment.

## Results

We begin our analysis of the experimental data by testing the two preliminary hypotheses that consider the effects of game characteristics and individual characteristics on performance.

We start from hypothesis **H0.A**, which considers the effects of the treatment variables on performance. Recall, from Section 3.3, that the three measures of performance in our experiment are: number of hands, moves/hands ratio, and time/hands ratio. A subject’s better performance in the TTT game is characterized by higher number of hands and lower moves/hands ratio and time/hands ratio.

First, we note that training a subject on 422 or on 442 makes no significant difference in terms of performance. Indeed, the average number of hands (24.26 vs. 23.73, *p*-value = 0.845), the average moves/hands ratio (15.14 vs. 12.09, *p*-value = 0.568) and the average time/hands ratio (125.88 vs. 98.34 seconds, *p*-value = 0.755) are not significantly different between treatments according to the strategies subjects have been trained to (Mann-Whitney test of equality of medians between two independent sampled populations). Hence, **H0.A.i is verified**.

We now move to compare the treatments with fixed goal and the treatments with changing goal. As discussed in Section 3.3, we expect subjects in treatments with changing goal to have on average worse performances. Specifically, we find that treatments with changing goal have lower average number of hands (23.10 vs. 24.87, *p*-value = 0.013), higher moves/hands ratio (14.81 vs. 12.49, *p*-value = 0.002), and higher time/hands ratio (114.69 vs. 109.86 seconds, *p*-value = 0.002) than treatments with fixed goal. Hence, **H0.A.ii is verified**.

Finally, we consider the effect of covering the opponent role’s cards. Consistently with H0.A.iii, we find a higher moves/hands ratio in covered treatments than in uncovered ones (14.16 vs. 13.04), but the difference is not significant (*p*-value = 0.774). Again consistently with H0.A.iii, we detect a significantly lower average time/hands ratio in covered than uncovered treatments (97.94 vs. 127.83, *p*-value < 0.001). Recall that H0.A.iii is silent as for which of the two opposite effects – higher moves/hands ratio vs. lower time/hands ratio – would prevail. Having detected only the second effect to be significant, which goes in the direction of a better performance by covered treatments, it is not surprising that the average number of hands is significantly higher in covered than uncovered treatments (25.60 vs. 22.26, *p*-value < 0.001). This leads us to conclude that in the (less difficult) uncovered treatments subjects complete a lower number of hands because they think for a longer time, and hence they are slower. With this, we conclude that **H0.A.iii is mostly verified**.

All in all, we can conclude that **H0.A is verified**, with the exception of a non-significant difference in the moves/hands ratio between covered and uncovered treatments, because the expected positive difference is not a statistically significant one.

We now proceed to test H0.B. Consistently with H0.B.i, we find that age has a slight negative effect on subject’s performance. In fact, the Spearman’s rank correlation index is significantly negative between age and number of hands ($-0.09$, *p*-value = 0.055), non-significant between age and moves/hands ratio (*p*-value = 0.304) and significantly positive between age and times/hands ratio (0.10, *p*-value = 0.047). Hence, age has mostly the expected effect on performance.

As for the effect of education on performance, results are only partially in line with our hypothesis. In fact, we report a significantly negative effect on moves/hands ratio ($-0.11$, *p*-value = 0.023) and essentially no effect on number of hands and time/hands ratio (respectively, 0.04, *p*-value = 0.392; $-0.02$, *p*-value = 0.620).

The effects of gender, instead, are the expected ones. Indeed, females complete a lower number of hands (22.85 vs. 24.69) with higher moves/hands ratio (13.78 vs. 13.72) in a higher time/hands ratio (118.31 vs. 111.65 seconds), all these differences being highly significant (*p*-value equal respectively to 0.019, 0.004, 0.003).

Finally, as for inebriation, we detect no correlation at all between subjects’ BAC and their performance in the TTT game (Spearman’s rho = 0.02, *p*-value = 0.724 for number of hands; Spearman’s rho = 0.01, *p*-value = 0.810 for moves/hands ratio; Spearman’s rho = 0.00, *p*-value = 0.990 for time/hands ratio). We find a slight negative correlation if we focus on inebriated subjects (i.e., with BAC > 0), but the correlation index is usually not significant: Spearman’s rho = $-0.14$, *p*-value = 0.142 for number of hands; Spearman’s rho = 0.21, *p*-value = 0.025 for moves/hands ratio; Spearman’s rho = 0.12, *p*-value = 0.223 for time/hands ratio. This result is confirmed by the fact that no significant difference in performance is found between sober (i.e., with BAC = 0) and inebriated subjects, regardless of the measure of performance (lowest *p*-value = 0.553 for average number of hands). Therefore, inebriation does not have the effect predicted by H0.B. We consider this result as relevant for the following analysis, since it allows us to consider the whole sample of participants – rather than the sub-sample of sober ones – when testing our experimental hypotheses on learning and routinization.

All in all, we can conclude that **H0.B is mostly verified**, with the exception of the effects of inebriation on performance.

For the sake of confirmation of the previous non-parametric tests of H0, Table 2 below reports the results of three OLS regressions, with respectively number of hands, moves/hands ratio, and time/hands ratio as dependent variable, and with the treatment manipulations of H0.A and idiosyncratic features of H0.B as explanatory variables. The parametric analysis essentially confirms the results of the above non-parametric testing. As for H0.A, the strategy the subject has been made familiar with (422 or 442) has no significant effect on any measure of performance (strategy 422 is taken as control in Table 2). Considering as control treatment the one with fixed goal and covered cards, the interaction between the two treatment manipulations confirms that: the negative effect of changing goals on the number of completed hands and through the increase in the time/hands ratio is significant in the uncovered treatments; the effect of covered cards in increasing the moves/hands ratio is significant when the goal changes across hands. As for H0.B, the significantly negative effect of age on performance is confirmed for the three measures (education has been omitted because of 0.61 rank correlation with age, *p*-value < 0.001). The detected negative effect of gender on performance is found significant only for the number of hands, while the non-significant effect of inebriation is confirmed regardless of the measure of performance.

**Table 2:** OLS regression on the three measures of performance (H0)

	No. of hands	Moves/hands	Time/hands
Strategy 442	–1.571	–2.144	–4.185
	(0.982)	(2.174)	(19.975)
Changing & Uncovered	$$-2.889^{**}$$	3.490	$$62.687^{*}$$
	(1.433)	(3.175)	(29.171)
Fixed & Covered	1.418	3.558	$48.973^{*}$
	(1.407)	(3.116)	(28.629)
Changing & Covered	0.436	$5.594^{*}$	13.487
	(1.424)	(3.155)	(29.988)
Age	$$-0.265^{***}$$	$$0.387^{***}$$	$$8.198^{***}$$
	(0.062)	(0.137)	(1.259)
Female	$-1.831^{*}$	0.861	18.119
	(0.999)	(2.212)	(20.325)
BAC (alcohol test)	–1.560	–7.634	–64.46
	(2.692)	(5.964)	(54.793)
Constant	$$34.196^{***}$$	1.650	$$-136.337^{***}$$
	(2.491)	(5.519)	(50.700)
Observations	418	418	418

* *p*-value $< 0.1$, ** *p*-value $< 0.05$, *** *p*-value $< 0.01$

We now turn to the analysis of learning in the training phase and routinization in the second phase of the TTT game, by testing our main hypotheses H1-H3. First, let us recall that learning in the training phase is captured by a dummy variable that classifies a subject as *expert* (value 1) if he chooses the right contextual strategy (according to treatments 422 or 442) in at least $75\%$ of the hands he plays out of the first 16 (i.e, in the training phase), and *inexpert* (value 0) otherwise. We also recall that routinization and creativity in the second phase are captured by a dummy variable that classifies a subject as *routinized* (value 1) (resp., *creative*, value 0) if he chooses, in at least (resp., less than) $75\%$ of the hands he plays in the second phase of the experiment, the contextual strategy he has been made familiar with in the training phase (i.e., according to treatments 422 or 442).^8^

With this, we can test **H1** by relying on the two dummy variables *expert* and *routinized*. The detected share of *expert* subjects is low (10.09%), and significantly higher in 422 than in the 442 treatments (14.35% vs. 5.75%, *p*-value = 0.002, $\chi ^2$ test). We interpret this finding as a signal that strategy 442 is more difficult to learn, so that subjects needed a higher number of hands to converge on it. However, and possibly for the same reason, once they have learned strategy 442, they routinize on it more strongly. In fact, we note that the fraction of subjects completing more than 16 hands of the game (i.e., overcoming the training phase) is not significantly different between the 422 and the 442 treatments (75.22% vs. 70.35%, *p*-value = 0.243, $\chi ^2$ test). However, among those who reached at least hand 17, we find a significantly lower share of *routinized* subjects in 422 than in the 442 treatments (19.08% vs. 34.59%, *p*-value = 0.001, $\chi ^2$ test).

**Fig. 4:**
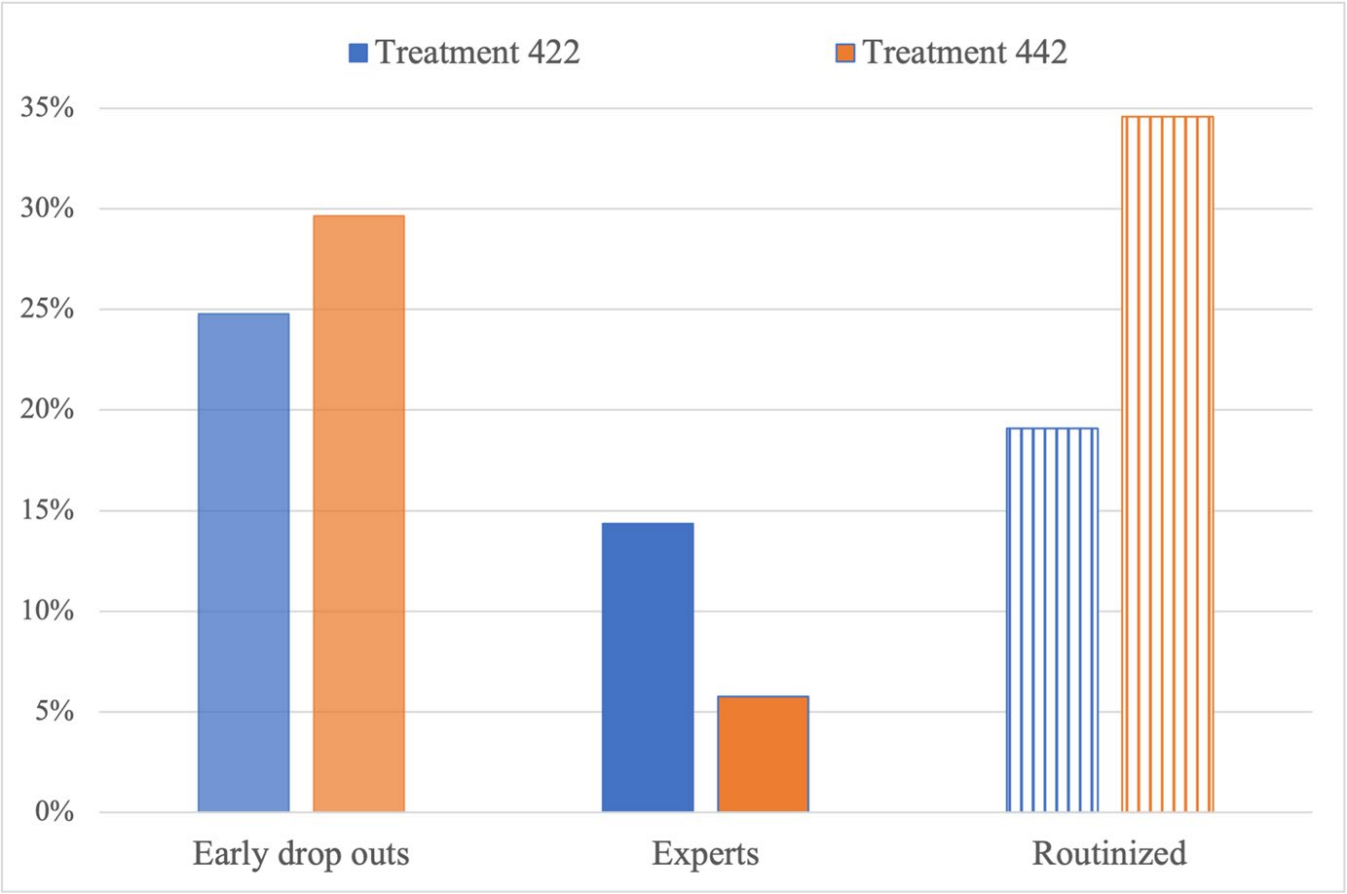
Share of early drop out, expert and routinized subjects in 422 vs. 442 treatments

With this, we can state that **H1.i is not verified**. This is represented in Fig. 4, where we report the share of subjects not overcoming the training phase (namely, *early drop out* subjects – histograms in lighter color), the share of experts (histograms in darker color) and the share of routinized subjects (histograms with linear shape fill). Note that routinized subjects belong to the complementary sub-sample of subjects (not early drop outs) who did overcome the training phase. H1.i is not verified since, despite a similar share of early drop outs in 422 and 442 treatments, the share of expert (resp., routinized) subjects is significantly higher in treatment 422 (resp., 442).

**Fig. 5:**
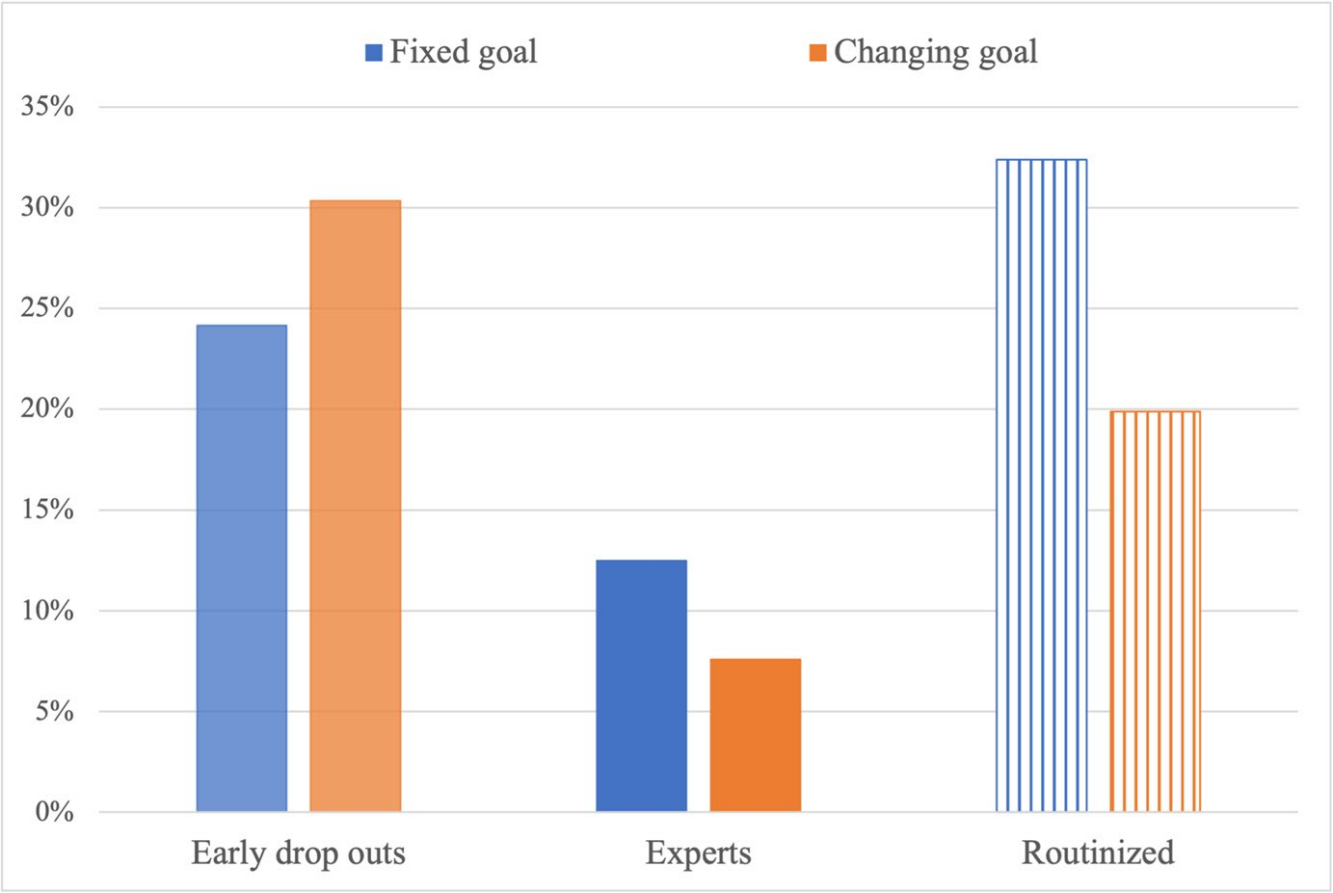
Share of early drop out, expert and routinized subjects in fixed vs. changing goal

We now turn to the second treatment variable, by comparing treatments with fixed goal to treatments where the goal card is changing. The hypothesis that the share of expert subjects is higher in treatments with fixed goal than in treatments with changing goal is verified (12.50 vs. 7.59, *p*-value = 0.082). The hypothesis that the share of routinized subjects is higher in treatments with changing goal than in treatments with fixed goal is instead not verified. As a matter of fact, the share of routinized subjects is significantly higher in fixed than in changing treatments (32.39 vs. 19.87, *p*-value = 0.010). As such, H1.ii is verified for learning but contradicted for routinization, as it is shown in Fig. 5 (same color code as Fig. 4), which also reports a non-significantly different share of early drop outs in the two treatments (*p*-value = 0.244). However, we recall that, given the nature of the TTT game, we can only observe subjects’ routinization if they managed to learn the routine in the training phase. We believe that the explanation of the comparison between fixed and changing treatments has to be found in the fact that the changing treatment is so complicated that only few subjects managed to learn the correct contextual strategy in the training phase, as it is shown by the negligible share of expert subjects (less than 8%) and the high share of early drop outs (more than 30%) in the treatments with changing goal.

**Fig. 6:**
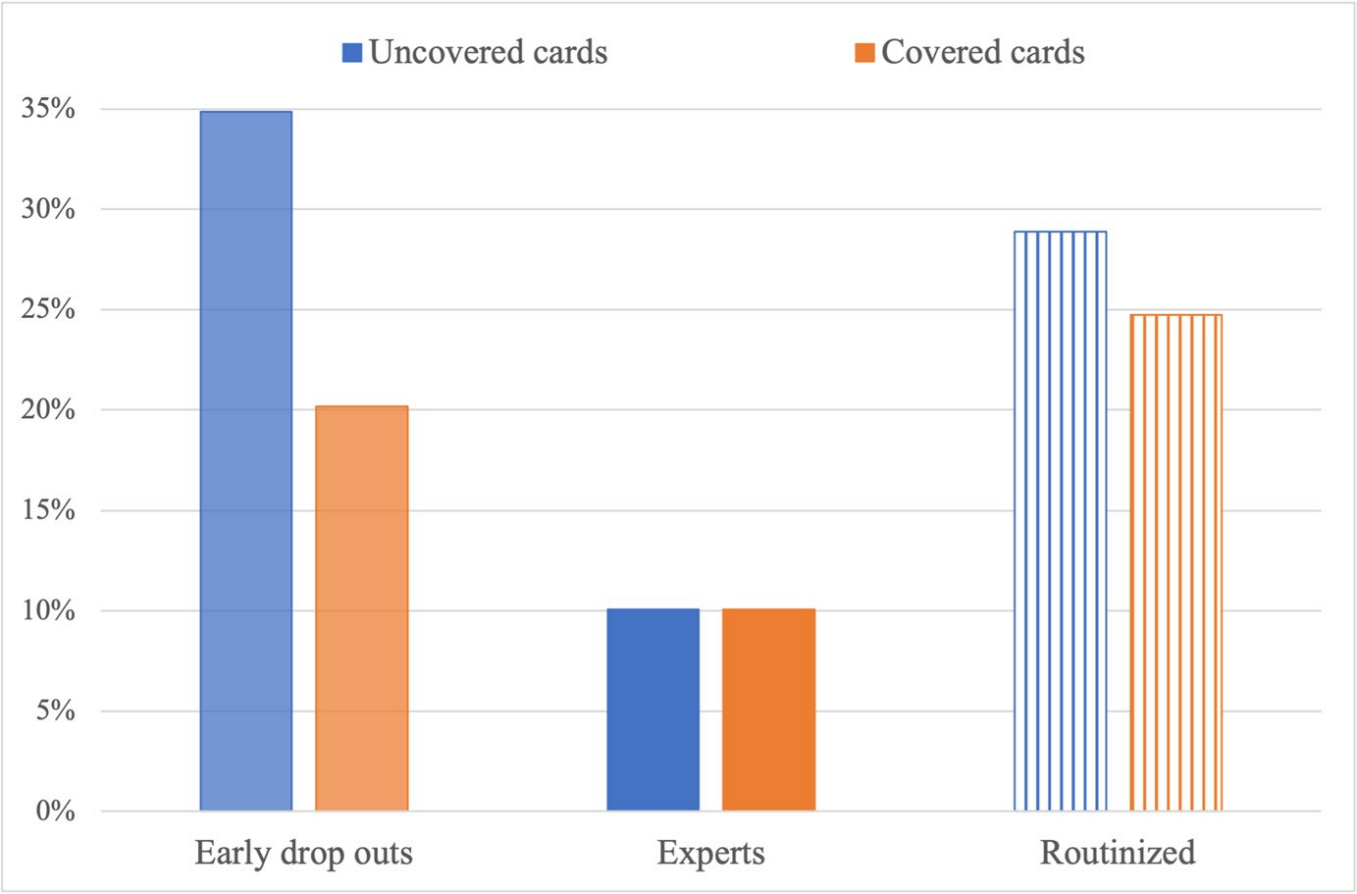
Share of early drop out, expert and routinized subjects in uncovered vs. covered cards

Finally, let us investigate the effects of the last treatment variable on learning and routinization, by comparing treatments where the opponent role’s cards are covered to treatments where they are uncovered. Figure 6 (same color code as Figs. 4-5) shows the same (low) share of expert subjects between uncovered and covered treatments (10.09 vs. 10.08, *p*-value = 1.000), and a non-significant difference in the share of routinized subjects (28.87 vs. 24.74, *p*-value = 0.398). Hence, we conclude that H1.ii is not verified as for the difference between covered and uncovered treatments. This confirms our findings on performance (test of H0.A.iii), which suggested that we cannot conclude that covered treatments are cognitively more complicated than uncovered ones, as, on the one hand, they make it harder to figure out the optimal strategy but on the other hand they require the subject to process less information. And in fact, as Fig. 6 shows, the share of early drop outs is significantly higher in uncovered than in covered treatments (34.86% vs. 20.17%, *p*-value < 0.001). The last result highlights the higher cognitive load required by uncovered treatments.

We complement the test of H1 with logit regression models predicting whether a subject is respectively an early drop out, an expert, a routinized, or an early finisher, i.e., completing the 32 hands of the game before the allowed 25 minutes. The last dependent (time) variable – which is not represented in Figs. 4-6 – has been introduced in order to capture a further dimension of the learning and routinization processes at work: time management. For each of the four binary variables under scrutiny, Table 3 reports the odds ratios of the three treatment variables of H1 and of the subjects’ idiosyncratic features of H2.^9^ As we did in Table 2, we consider as control treatments strategy 422, fixed goal and covered cards, and we study the interaction between the last two treatment manipulations. Table 3 essentially confirms the results of the above non-parametric testing of H1. Indeed, letting subjects familiarize with the contextual strategy 442 significantly increases the likelihood of early drop outs and decreases the likelihood of learning the contextual strategy. However, if the contextual strategy is 442, then the likelihood of becoming routinized to it in the second part of the game significantly increases. The negative effects of the treatment with changing goal on learning and routinization are found significant conditionally on covered cards (odds ratio significantly lower than 1 for the Changing & Covered interaction in both columns “Experts” and “Routinized”), while its negative effect on finishing the game in advance is found significant conditionally on uncovered cards (odds ratio significantly lower than 1 for the Changing & Uncovered interaction in column “Early finishers”). No significant effect is detected for the covered-card manipulation, again in line with the previous non-parametric analysis. With this, we can conclude that **H1.ii is only partially verified**.

**Table 3:** Logistic regression on learning and routinization (H1-H2)

	Early drop outs	Experts	Routinized	Early finishers
Strategy 442	$$1.558^{*}$$	$$0.273^{***}$$	$$2.380^{***}$$	0.918
	(0.365)	(0.104)	(0.665)	(0.190)
Changing & Uncovered	1.571	0.942	0.838	$$0.542^{**}$$
	(0.500)	(0.462)	(0.339)	(0.164)
Fixed & Covered	0.634	1.441	1.015	1.604
	(0.218)	(0.662)	(0.374)	(0.478)
Changing & Covered	0.881	$$0.341^{*}$$	$$0.388^{**}$$	0.939
	(0.291)	(0.217)	(0.164)	(0.277)
Age	$$1.040^{***}$$	0.981	0.975	$$0.966^{**}$$
	(0.014)	(0.021)	(0.022)	(0.013)
Female	1.350	0.558	0.756	$$0.543^{***}$$
	(0.314)	(0.212)	(0.216)	(0.115)
BAC (alcohol test)	1.338	$0.046^{*}$	0.508	0.405
	(0.830)	(0.075)	(0.456)	(0.232)
Constant	$$0.073^{***}$$	0.805	0.788	$$6.666^{***}$$
	(0.043)	(0.670)	(0.621)	(3.591)
Observations	418	418	418	418

Odds ratio are reported; * *p*-value $< 0.1$, ** *p*-value $< 0.05$, *** *p*-value $< 0.01$

We now proceed to test **H2**, i.e., the potential correlations between learning and routinization on the one side and subjects’ idiosyncratic features on the other side. We detected a slight negative effect of age on routinization (Spearman’s rho = $-0.10$, *p*-value = 0.068), while no effect was found for learning (Spearman’s rho = $-0.02$, *p*-value = 0.737). Therefore, it seems that an older age leads to more routinization in the second phase: once learned the right contextual strategy, older subjects stuck to it more, without trying anything different in the hands where this was needed to reduce the number of moves. As expected, education had no effect on either learning or routinization (respectively, Spearman’s rho = $-0.01$, *p*-value = 0.961; $-0.01$, *p*-value = 0.886). Also gender had no effect on routinization in the second phase ($\chi ^2$ test, *p*-value = 0.376), while males showed significantly more learning than females in the training phase (11.99% vs. 6.43%, *p*-value = 0.057, $\chi ^2$ test), which is consistent with their better performance (test of H0.B).

As for the effect of inebriation, the correlation between BAC and both learning and routinization is negative (respectively, Spearman’s rho = $-0.08$ and $-0.10$) and significant only for routinization (respectively, *p*-value = 0.103 and 0.077). Focusing only on inebriated subjects, we find again a negative correlation of BAC with both learning and routinization (respectively, $-0.16$ and $-0.09$), significant only for the former (respectively, *p*-value = 0.092 and 0.406). A Mann-Whitney test confirms that inebriated subjects routinized significantly less than sober ones in the second phase of the game (19.77% vs. 29.09%, *p*-value = 0.097). They also made less learning in the training phase, but not significantly so (6.36% vs. 11.04%, *p*-value = 0.158).

All in all, we can conclude that **H2 is mostly verified**. Indeed, the logit regression models in Table 3 confirm no systematic effect of idiosyncratic features on learning and routinization (second and third column) with the exception of the negative effect of inebriation on learning. However, although not having significant effects on the cognitive process, age and gender do impact on the game outcome: Table 3 shows that being older significantly increases the likelihood of dropping out early and significantly decreases the likelihood of finishing the game early, the latter being the case also for female subjects.

We conclude the analysis with the test of **H3**, which deals with the performance of expert vs. inexpert subjects in the training phase (H3.i) and of routinized vs. creative subjects in the second phase (H3.ii). **H3.i is strongly verified**: expert subjects completed a significantly greater number of hands than inexpert ones (31.37 vs. 23.18, *p*-value < 0.001), and presented a significantly lower moves/hands ratio (6.04 vs. 14.48, *p*-value < 0.001) and time/hands ratio (32.12 vs. 121.22 seconds, *p*-value < 0.001).

The fact that expert subjects perform better than inexpert ones in terms of (lower) moves/hands ratio informs us that inexpert subjects are kind of lost in their effort to find the right strategy in the training phase, this strategy being the same regardless of the first 16 hands of the game. For this reason, when testing H3.ii as for the effect of routinization on performance, we disentangle expert and inexpert subjects. With this, we rely on four mutually-exclusive categories of players: inexpert creative (67.47% of the sample), inexpert routinized (26.72% of the sample), expert creative (8.62% of the sample), and expert routinized (11.21% of the sample). These four categories disentangle the sub-sample of subjects overcoming the 16-hand training phase, i.e., completing at least 17 hands of the game (72.81% of the sample). The residual category of early drop outs in Figs. 4-6 (27.19% of the sample) is not considered in the test of H3.ii.

**Fig. 7:**
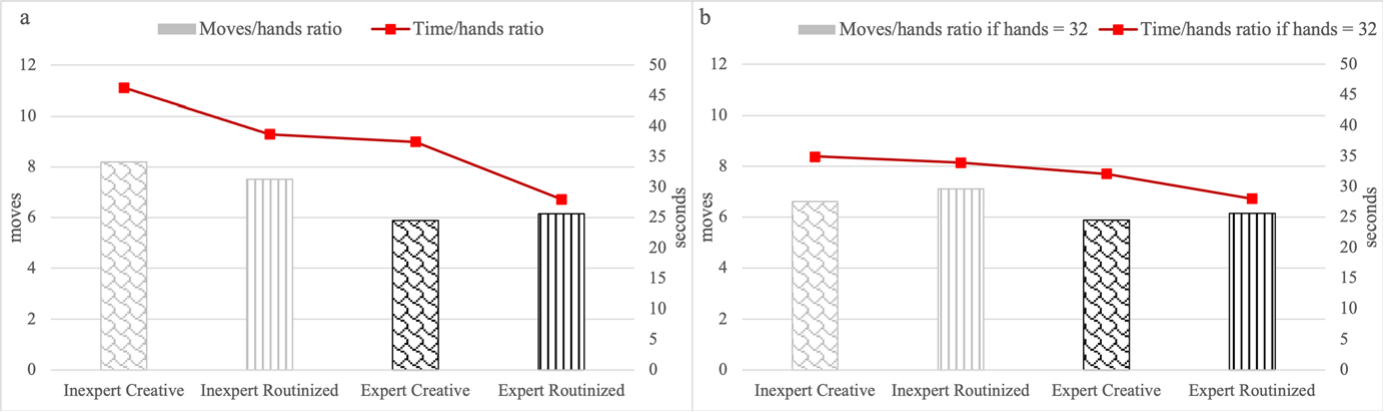
Performance of creative vs. routinized subjects, disentangled by expertise

Figure 7a reports two out of the three measures of performance of the four categories of subjects overcoming the 16-hand training phase, namely moves/hands ratio and time/hands ratio. Figure 7b reports the same two measures for the same four categories of subjects, conditionally on having completed the whole 32 hands of the TTT game. The color code of histograms in the figure is the same as in Figs. 4-6, with inexpert subjects having the same color code as early drop outs in Figs. 4-6 (histograms in lighter color) as compared to expert ones (histograms in darker color), and creative subjects represented through histograms with non-linear shape fill as compared to routinized ones (linear shape fill). We highlight that the sub-sample of subjects in Fig. 7b is modal among those subjects overcoming the training phase. In fact, 62.95% of inexpert creative, 83.87% of inexpert routinized, 90% of expert creative and 100% of expert routinized completed the 32 hands of the TTT game.

From Fig. 7a it is easy to notice that, among inexpert subjects, routinized subjects perform significantly better than creative ones in terms of lower time/hands ratio (38.72 vs. 46.29, *p*-value = 0.001), which confirms H3.ii, but not significantly worse in terms of higher moves/hands ratio (7.52 vs. 8.19, *p*-value = 0.291), which is inconsistent with H3.ii. Given that the first effect is predominant, inexpert routinized subjects complete a significantly higher number of hands than inexpert creative ones (30.77 vs. 28.95, *p*-value = 0.002). If we focus on subjects completing the whole TTT game (Fig. 7b), H3.ii is verified for moves/hands ratio but not for time/hands ratio: inexpert routinized and inexpert creative subjects show a similar time/hands ratio (33.94 vs. 34.97, *p*-value = 0.001) and the former performs significantly worse in terms of higher moves/hands ratio (7.11 vs. 6.61, *p*-value = 0.096). With this, we conclude that **H3.ii is partially verified for inexpert subjects**.

Moving to expert subjects, Fig. 7a shows that routinized subjects perform significantly better than creative ones in terms of lower time/hands ratio (28.01 vs. 37.46, *p*-value = 0.015), and worse in terms of higher moves/hands ratio, although not significantly so (6.16 vs. 5.88, *p*-value = 0.258). Both results are consistent with H3.ii, and are confirmed if one focuses on expert subjects completing the whole TTT game (Fig. 7b): the positive gap for time/hands ratio and negative gap for moves/hands ratio are respectively similar to those detected in the general case of Fig. 7a, with only the first gap being significant (respectively, *p*-value equal to 0.045 and 0.272). With this, we conclude that **H3.ii is verified for expert subjects**.

For the sake of confirmation of the non-parametric tests of H3, Table 4 below reports the results of five OLS regressions, with each of the three measures of performance – number of hands, moves/hands ratio, and time/hands ratio – as dependent variable in respectively columns 1, 2-3 and 4-5. Columns 2 and 4 complement the analysis of Fig. 7a. Columns 3 and 5 complement the analysis of Fig. 7b, i.e., we restricted the sample to subjects having completed the whole 32 hands of the TTT game. The explanatory variables are the dummies “Expert” and “Routinized” and the interactions among them (inexpert-creative being the baseline category). We included in the list of regressors the subjects’ idiosyncratic features as controls. The model in column 1 confirms that routinization significantly increases the number of completed hands regardless of acquired expertise in the first phase of the game: the latter only has a positive effect on the number of hands if combined with routinization in the second phase of the game. The model in column 4 shows that the results on the time/hands ratio mirror those on the number of completed hands (column 1). However, if one focuses on subjects completing all the 32 hands (i.e., the potential winners of the tournament), only the interaction between expertise and routinization significantly decreases the time/hands ratio. Furthermore, the model in column 2 confirms that acquired expertise significantly decreases the moves/hand ratio, regardless of routinization in the second phase of the game. The latter contributes to decrease the moves/hand ratio only for expert subjects. Finally, and more importantly, focusing on those subjects completing all the 32 hands of the game (column 3, the potential winners), routinization in the second phase of the game significantly contributes to an increase in the moves/hand ratio for inexpert subjects.

**Table 4:** OLS regression on performance of experts and routinized (H3)

	No. of hands (h)	Moves/h	Moves/h 32	Time/h	Time/h 32
Inexpert & Routinized	$$1.399^{**}$$	–0.547	$$0.457^{*}$$	$$-5.864^{**}$$	–0.855
	(0.651)	(0.497)	(0.266)	(2.431)	(1.176)
Expert & Creative	0.995	$$-1.921^{**}$$	–0.550	–6.149	–2.464
	(1.078)	(0.823)	(0.417)	(4.026)	(1.842)
Expert & Routinized	$$2.723^{***}$$	$$-1.746^{**}$$	–0.404	$$-17.073^{***}$$	$$-7.036^{***}$$
	(0.960)	(0.733)	(0.355)	(3.585)	(1.571)
Age	–0.401	$$-0.720^{**}$$	–0.025	0.171	0.057
	(0.039)	(0.030)	(0.017)	(0.145)	(0.075)
Female	–0.706	–0.107	0.054	$$4.090^{**}$$	$$2.099^{**}$$
	(0.524)	(0.400)	(0.230)	(1.956)	(1.016)
BAC (alcohol test)	–1.330	$$2.891^{**}$$	0.797	4.640	–1.287
	(1.482)	(1.131)	(0.656)	(5.533)	(2.897)
Constant	$$31.222^{***}$$	$$9.571^{***}$$	$$6.995^{***}$$	$$35.333^{***}$$	$$30.823^{***}$$
	(1.268)	(0.968)	(0.551)	(4.737)	(2.434)
Observations	306	306	222	306	222

32 stands for “if all 32 hands completed”; * *p*-value $< 0.1$, ** *p*-value $< 0.05$, *** *p*-value $< 0.01$

All in all, our test of H3 seems to show that learning the suggested contextual strategy in the training phase generates a significant gap in the performance in the second phase (H3.i). However, for expert subjects, routinizing in this (unique) strategy has no impact on the number of completed hands, and decreases performance as for moves/hands ratio, although not significantly so. Furthermore, for inexpert subjects, although significantly increasing the number of completed hands, routinization has no impact on the moves/hands ratio and, among those subjects completing the whole game, it even has a significant negative impact on the moves/hands ratio. In one sentence, given that 71.39% of our subjects overcoming the training phase were able to complete the whole game, routinization decreased overall the likelihood to win the game (H3.ii). Indeed, in each of the 24 sessions of our TTT game, what counted for the final ranking was the moves/hands ratio, since usually more than half of the subjects in a session completed the 32 hands (51.97% on average across all sessions).^10^

## Conclusions

Understanding the process by which individuals routinize, and whether routinization is efficient in completing difficult tasks is relevant for most organizations.

In the paper, we tackle this issue through a lab-in-the-field experiment in which we first train a subject to solve a decision problem, the TTT game in its puzzle form, with one optimal strategy, and then we propose to the same subject different versions of the puzzle where the strategy that was used in the training phase may no longer be optimal. Our analysis focuses on this second phase of the game, and on the effects of a creative approach vs. routinization.

Our experiment is built to test the hypothesis that by exposing players to a set of preliminary hands characterized by starting configurations all easily solved by the same strategy, they would be “induced” to discover this solution more easily than the alternative one and to memorize it more deeply. This mechanism is strengthened by exogenously imposing time pressure, which discourages subjects from exploring the strategy space, and therefore favors routinization.

We find that, although younger and male subjects show a better performance in the TTT game, routinization and learning are mostly independent of idiosyncratic features of individuals.

As for the efficiency of routinization, we find that the effects of routinization on performance depends on whether subjects are expert or not. Notably, we find that for expert subjects there is no clear difference between the performance of routinized and creative subjects: the former are faster, the latter take fewer moves to complete a hand. A difference is instead present in those subjects who were slow at learning the correct strategy in the training phase. Among inexpert subjects, we find that routinized subjects are faster, so that their average number of completed hands is higher than the creative subjects’ one, but so is the moves/hands ratio if completing the 32 hands of the game. Overall, this reduces their likelihood of winning the game, as most winners tied in terms of number of hands (completing the maximum number of 32), so that the winner was selected according to the lower moves/hands ratio criterion.

In the paper, we also look at three treatment manipulations of the TTT game: type of suggested strategy in the training phase (422 vs. 442), fixed vs. changing goal, and uncovered vs. covered opponent role’s cards. Among the treatment effects, there is one which represents a particularly interesting test of creativity, namely the fixed vs. changing goal treatment. As a matter of fact, in the presence of a changing goal, both in the learning phase and in the routinization phase subjects, in order to replicate the same strategy for a different goal, need to change the representation of the problem and of the strategy itself. This is in itself a higher form of creativity, which creates a new and richer structure of rules in the mind of subjects, in the spirit of Simon (1969, pp. 94-98). In this interpretation, learning in the training phase of the changing goal treatment may itself be considered creative, and is therefore more difficult than learning in other treatments, which is consistent with our findings.

Our puzzle version of the TTT game was designed with the aim of isolating learning and routinization that are not related to coordination issues but rather to the cognitive individual process. The individual decision-making environment that this simplified version of the TTT game involves makes it appropriate for an online experimental setting. Throughout the paper, we discussed the reasons why we opted for a lab-in-the-field experiment. First, framing our experiment as an event of a mass gathering festival, and advertising it in local newspapers and TV news some days before the festival, and across the festival through posters, flyers, and announcements on social networks, made it easier to recruit almost 500 subjects within 2 days. This allowed us to run all experimental sessions under the same environmental conditions, and at the same time guaranteed high population size and heterogeneity. Furthermore, the naturalistic environment of the festival made the experiment seem as a game of competition with cards, in the true spirit of the Target-The-Two game of Cohen and Bacdayan (1994).

We performed our lab-in-the-field in August 2017, i.e., long before the COVID-19 pandemics, which has dramatically increased the frequency of online experiments, with researchers significantly improving techniques to recruit representative subject pools, implement video controls during the experiment, and perform payment procedures credible to subjects (see, e.g., Buso et al. 2021). At the same time, by replicating traditional laboratory experiments, experimental economists have shown that lab-like findings from online experiments are not so far from those previously found in the laboratory (see, e.g., Buso et al. 2020). Thanks to these new techniques, which involve online visually monitored sessions (e.g., through Cisco WebEx or Zoom), and thanks to the fact that our TTT game software rests on an online platform (Graphgames), it is nowadays possible to safely replicate our field experiment online. An online experiment, by dramatically increasing the number of observations, could provide support to the internal and external validity of our study. With this, we suggest the implementation of the online TTT puzzle-game experiment and the comparison between field and online data as an extension of the study in this paper.

## Data Availability

The raw data supporting the conclusions of this article will be made available by the authors, without undue reservation.

## Footnotes

1 As an example of such artefactual field experiment, Harrison and List (2004) discuss the paper by Harrison et al. (2002) who use a standard lab experiment but instead of running it at a university they run it in hotels in order to be able to attract a representative sample of the Danish population.

2 For a comprehensive analysis of the relevant literature on the TTT game, see Becker (2001, 2004).

3 See Attanasi et al. (2021a) for a systematic review of creativity tasks, categorized as “closed” (in box: with ex-ante goals and constraints), “open” (blue sky: without ex-ante goals and constraints), and “open with constraints”﻿ (without ex-ante goals but with constraints), at a middle point between the first two classical categories.

4 The problem of the switch from routinized to exploratory behavior has been studied also at the level of neural activity. Schuck et al. (2015) show through multivariate neuroimaging analyses that, once a given strategy has become established, the medial prefrontal cortex tends to inhibit those elements that could bring about an alternative categorization of the problem, making it more difficult for the agent to adopt any innovative strategy. Exploration is hindered by our own devices, yet when we adopt exploratory behavior, we do so before we become conscious of its adoption.

5 The dualism between the automatic process of recall and the effortful process of symbol manipulation has been deeply explored (see Shiffrin and Schneider 1977, and Kahneman’s Nobel lecture). The dual view is based on the evidence that a large part of neural activity is related to automatic processes, which are faster than conscious deliberations and which occur with little or no awareness of effort. As within Simon’s (1969) analysis, thinking is supposed to be composed of two different cognitive processes: on the one hand a controlled, deliberate, sequential, and effortful process of mental manipulation of items; on the other hand, a non-deliberate, automatic, effortless, and fast process of eliciting mental items from long-term memory.

6 The same type of electronic breathalyzers with the same technique were used to measure BAC for other field studies run during the same event (see Attanasi et al. 2017, 2021c).

7 Due to technical failure (failed data recording during some experimental sessions), we turned out to have a different number of subjects per treatment, and we had to re-run one session entirely (with new subjects). The average number of subjects with no technical problem per session is 57 (total number = 456), with their minimum number per session being 52.

8 Note that given our definition of routinized and creative subjects, we can only classify subjects as routinized or creative if they play at least 17 hands.

9 For binary choice variables in Table 3, the odds ratio is the ratio of the probability that a specific player’s type (e.g., expert) emerges in one treatment (e.g., strategy 442) or in one group (e.g., female) to the probability of it occurring in the complementary treatment or group (resp., strategy 422 or male). For ordinal variables in Table 3 (age and BAC) the interpretation of the odds ratio is similar to that of a linear regression coefficient: it indicates the average effect of a higher value of the variable (a higher age, a higher BAC), ceteris paribus, on the probability of a specific player’s type emerging in the game.

10 As one could expect, it never occurred that two subjects completing the 32 hands in the same session also had the same moves/hands ratio, hence a lower time/hands ratio was never useful to win the game.
